# A Historical Perspective and Update on Robotic Mitral Valve Surgery

**DOI:** 10.3390/jcm13216375

**Published:** 2024-10-24

**Authors:** Amy Chartrain, Alfredo Trento, George Gill, Dominic Emerson, Wen Cheng, Danny Ramzy, Joanna Chikwe

**Affiliations:** 1 Department of Cardiac Surgery, Smidt Heart Institute, Cedars-Sinai Medical Center, Los Angeles, CA 90048, USA; 2 Department of Cardiac Surgery, UTHealth Houston, Houston, TX 77030, USA; danny.ramzy@uth.tmc.edu

**Keywords:** robotic surgery, robotic mitral valve repair, minimally invasive cardiac surgery

## Abstract

**Background/Objectives**: Minimally invasive techniques for mitral valve repair have evolved over the past thirty years and include mini-thoracotomies and the robotic platform. This study provides a historical perspective on minimally invasive mitral valve approaches and evaluates long-term outcomes of a large series of robotic mitral valve repairs. **Methods**: A single-institution, prospectively maintained registry was used to evaluate robotic mitral valve repairs performed by four surgeons from 2005 to 2023. There were 1412 robotic mitral valve repairs performed during this time and stratified by the first 120 and subsequent patients. We evaluated operative outcomes and freedom from more than 2+ mitral regurgitation at five years as well as ten-year survival. **Results**: Of the 1412 robotic mitral valve repairs performed, 93.6% (*n* = 1322) were for degenerative disease. Compared to the first 120 patients, the subsequent patients had a significant reduction in cross-clamp time (112 (IQR = 103–130) versus 75 (IQR = 65–88) min) and cardiopulmonary bypass time (153.5 (IQR = 134.5–177.5) versus 116 (IQR = 103–136) min), and all with *p* < 0.01. The majority of patients had posterior leaflet prolapse (65.6%, *n* = 926). The repair rate was 98.1%, *n* = 1385. Survival at ten-year follow-up for the patients included in the first 120 procedures was 91.5% (95% confidence interval (CI) = 86.4–96.6%) versus 92.8% (95% CI = 91.7–93.9%) for the patients who were in the latter group, *p* = 0.58. Freedom from >2+ mitral regurgitation at 5 years was 97.0% (95% CI = 95.3–98.7%) for the patients in the first 120 procedures and 92.7% (95% CI = 91.5–93.9%), *p* = 0.22, for those in the latter group. **Conclusions**: The robotic platform offers an excellent durable repair for mitral regurgitation in our experience of over 1400 patients. The robotic platform for mitral valve repair offers a teachable and safe approach to mitral valve disease for patients.

## 1. Introduction

In the United States, minimally invasive cardiac surgery is now 30 years old. In 1995, HeartPort Inc., a start-up company based in Redwood City, CA, developed a technology that would allow coronary artery bypass to be performed through a left mini-thoracotomy incision promising less pain and trauma than the existing methods involving the median sternotomy [1]. John Stevens, a cardiac surgeon from Stanford and the co-founder of HeartPort, developed special catheters and other devices to perform the tasks necessary to complete minimally invasive surgery. A clinical trial involving ten patients was performed at Stanford University, opening the doors for the HeartPort platform to be introduced [2]. Since then, both in the United States and Europe, centers have developed minimally invasive techniques for coronary artery disease, aortic valve pathologies, and mitral valve repair.

Dr. Alain Carpentier introduced and popularized surgical techniques for mitral valve repair and the surgical pathophysiology and anatomy that has become a part of the learning curriculum of any surgeon [3]. Surgical pioneers adopted these techniques using mini-thoracotomies with femoral vessel cannulation. In Europe, Dr. Hugo Vanermen in Brussels and Dr. Fred Mohr in Leipzig developed significant experience and a program with endoscopic mitral valve repair [4,5]. In the United States, Dr. Steven Colvin and Dr. Gallaway at New York University had a similar experience and large series of patients [6]. We at Cedars-Sinai were also early adopters of this technology. In our experience, mitral valve repair through a mini-thoracotomy was patient-dependent. It was difficult in large patients, where the mitral valve looked far away from the surgeon’s view. The robotic platform was introduced in the early 2000s with a feasibility trial headed by Dr. Randall Chitwood at East Carolina University [7]. In 2005, we decided to start the program at Cedars-Sinai. The training involved spending a long weekend at East Carolina University where we observed Dr. Chitwood performing mitral valve repair, and then, we performed training on human cadavers. We also had a proctor who came to help us for the first three or four cases. The outcomes of the first 120 patients were reported to the Western Thoracic Surgical Association in 2010 and subsequently published in the *Journal of Thoracic and Cardiovascular Surgery* [8]. We were able to analyze our first 120 patients performed with the robotic technology available to us at that time. The visualization improved significantly after the first generation of the Da Vinci robot due to the improved left atrial retractor that was introduced. We noted that the improved outcomes may have been due in part to increased experience as well as the advancements in technology. As noted in the first analysis of the first 120 patients, there was one death and seven failed repairs that all occurred with the first subset of patients who had their surgeries with the older Da Vinci model. There was one mitral valve replacement in this first group of patients. We then subsequently reviewed and published an additional 180 patients for a total of 300 robotic mitral valve patients [9]. We noted a significant difference in outcomes between the two groups, notably the significantly lower cross-clamp and cardiopulmonary bypass times. This in part we attributed to the technological advances; however, it was noted that a second surgeon also performed part of these procedures, and despite the learning curve, we still had shorter operative times. Overall, 8 patients required subsequent replacements, and 2 of those occurred in the last 180 patients. We subsequently published our results of more than 1000 of our robotic mitral repairs. We noted the durable repair that this platform allowed, with freedom from >2+ mitral regurgitation at 10 years for isolated posterior prolapse to be 92% and 83% for anterior or bileaflet prolapse [10]. We also had good success with treatment of Barlow’s disease with this approach. We published our 111 patients over a 15-year time frame with survival free from greater than moderate mitral regurgitation to be 92% [11]. We now present our over 1400 robotic mitral repairs and compare them with our previously published initial cohort of 120 patients.

## 2. Materials and Methods

### 2.1. Data

A prospectively maintained institutional registry linked to a state-wide database was examined from 2005 to 2023. All patients over 18 years old were included in the study. The registry contained pre-operative, intra-operative, and post-operative details which were supplemented where needed by a retrospective chart review. Variables were defined according to The Society of Thoracic Surgeons Adult Cardiac Database and included versions throughout this time. The Institutional Review Board of Cedars-Sinai Medical Center approved this study (code of approval: STUDY00001188; approval date: 19 February 2021), which included a waiver of informed consent. 

Statistical analysis: All variables were checked for normal distribution, and where normally distributed, they are presented as mean and standard deviation, and where not normally distributed, variables are reported as median and interquartile ranges. Numeric variables were compared for patients in the first 120 procedures and those in the later procedures by *t*-test or Wilcoxon rank-sum test, depending upon their distribution. We compared categorical variables using chi-square test or Fisher exact test where appropriate. All statistical tests were two-tailed, and an alpha level of 0.05 was considered to be statistically significant. Statistical analysis was performed using SAS version 9.4 (SAS Institute, Inc, Cary, NC, USA).

### 2.2. Operative Technique

Over this time frame, four surgeons performed all operations. The second author (AT) performed these operations for the first 10 years and subsequently assisted other surgeons who began performing the operations. The described surgeries used two models of the Da Vinci robotic platform starting with the original model Da Vinci S in 2005 and subsequently the Da Vinci XI. The largest difference was that, in the first 74 cases, we did not have the atrial retractor as a separate articulating arm on the robot. The previous operative technique has been described in detail [8,10]. Briefly, at our institution, all robotic mitral repairs are performed as a two-attending procedure, and patients are evaluated for the appropriateness of the procedure based on a lack of significant aortic insufficiency, mitral annular calcification, peripheral arterial disease preventing peripheral cardiopulmonary bypass, and significant pulmonary dysfunction. All patients have had a mini-thoracotomy with peripheral cannulation, and we attempted to perform each repair where suitable with a triangular resection and flexible true-sized band as shown in Video S1. We did not exclude Barlow’s patients and have previously found this technique to provide excellent repair rates for these patients as shown in Video S2 [11]. The multiple degrees of freedom of the robotic arms also allows for the ease of suturing and the placement of neochordae, as shown in Video S3.

## 3. Results

**Figure 1:**
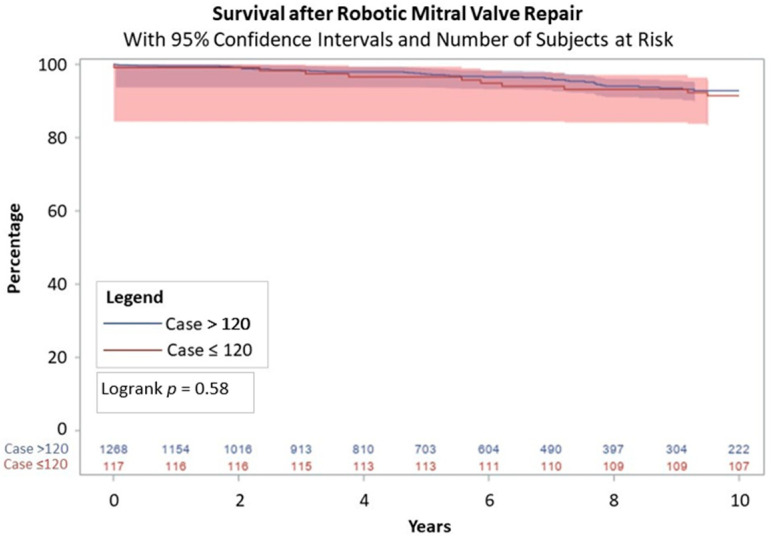
Ten-year survival after robotic mitral valve repair.

**Figure 2:**
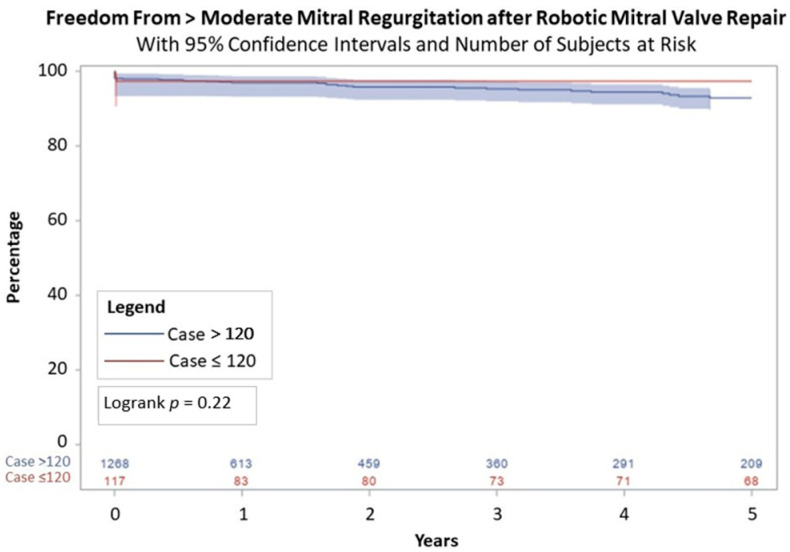
Five-year freedom from >2+ mitral regurgitation.

**Figure 3:**
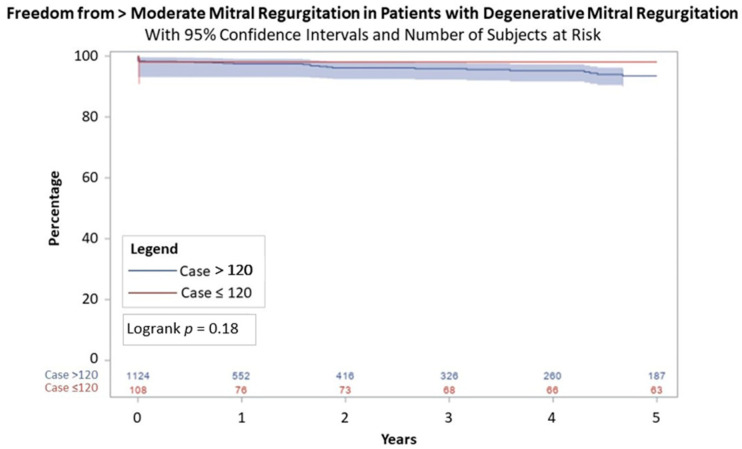
Five-year freedom from >2+ mitral regurgitation in degenerative disease.

From 2005 to 2023, there were 1412 robotic mitral surgeries performed by four operating surgeons. The median age was 63 years (interquartile range (IQR) = 54–70 years). Compared to the first 120 patients who had a median age of 56 years (IQR = 50–64 years), the median age increased in the later cohort with a median age of 63 years (IQR = 55–70 years), *p* < 0.01. The cohort was predominantly healthy with low incidences of diabetes (5.4%, *n* = 76), cerebrovascular disease (3.8%, *n* = 53), and prior myocardial infarction (2.8%, *n* = 39). Additional pre-operative characteristics including atrial fibrillation, LVEF, and STS PROM score are listed in Table 1. The majority of procedures were electively scheduled although there were 31 procedures classified as non-elective (Table 2). Compared to the first 120 patients, the subsequent patients had several differences noted in their operative characteristics. There was a significant reduction in cross-clamp time (112 (IQR = 103–130) versus 75 (IQR = 65–88) minutes) and cardiopulmonary bypass time (153.5 (IQR = 134.5–177.5) versus 116 (IQR = 103–136) minutes), and all with *p* < 0.01. The majority of patients had posterior leaflet prolapse (65.6%; *n* = 926). The repair rate was 98.1% (*n* = 1385). Operative mortality was 0.4% (*n* = 4) and included complications of vasoplegia, subclavian artery injury, pump failure, and suspected respiratory failure as shown in Table 3. The rate of reoperation for bleeding or cardiac etiology was 2.6% (*n* = 36). Length of stay significantly decreased after the first 120 patients to 5 days (IQR = 5–6 days) compared to 6 days (IQR = 5–7 days), *p* = 0.02. Survival at ten-year follow-up for the patients included in the first 120 procedures was 91.5% (95% confidence interval = 86.4–96.6%) versus 92.8% (95% CI = 91.7–93.9%) for the patients who were in the latter group, *p* = 0.58 (Figure 1). Freedom from >2+ mitral regurgitation at 5 years was 97.0% (95% CI = 95.3–98.7%) for the patients in the first 120 procedures and 92.7% (95% CI = 91.5–93.9%), *p* = 0.22, for those in the latter group (Figure 2). As shown in Figure 3, freedom from >2+ mitral regurgitation at 5 years for those with degenerative disease was similar between groups (97.9% (95% CI = 94.9–100%) versus 93.4% (95% CI = 91.0%−95.8%), *p* = 0.18). 

## 4. Discussion

Surgical approaches to mitral valve repair include median sternotomy and right lateral mini-thoracotomy with or without the Da Vinci Robotic System. The switch from a median sternotomy to a right mini-thoracotomy with robotic assistance was made on the basis of the following: (1) similar or improved visualization of the mitral valve and especially of the subvalvular apparatus; (2) the ability to perform any type of repair including anterior leaflet prolapse or flail using artificial cordae with extreme ease; (3) faster overall recovery from the surgical procedure, especially in patients over age 70; and (4) improved patient acceptance, because of the small scar often hidden under the breast. Our experience with the robotic platform to treat mitral valve disease of over 1400 patients has demonstrated that the technique has reproducible, excellent long-term outcomes with low rates of replacement or recurrence from >2+ mitral regurgitation. Our results of freedom from >2+ mitral regurgitation or replacement are similar to other published centers [12]. Additionally, compared to national data evaluating outcomes of robotic mitral valve surgery, we have found similar rates of post-operative complications [13]. We have found many benefits to the robotic platform since its adoption as previously mentioned. Additionally, with a two-surgeon approach, we have had excellent outcomes and low conversion rates. The two-surgeon approach also allows us to use another console for a learner, which with the robot allows for the ability to have the same visual field as a learner and control over the instruments, which is not available on any other platform. We believe this will improve the ability of younger surgeons and trainees to learn mitral valve surgery. Complications were comparable or better than published rates with those undergoing a median sternotomy approach. We had 10 strokes, 4 in the first 120 patients, and 6 in the subsequent cases, with an incidence of 0.5% in the latter group. We have not seen a stroke in the recent several hundred cases. We believe that avoiding the dissection of the interatrial groove fat is one reason for this. As no stroke was seen in the recent several hundred patients, this has resulted in great benefit for a typically more elderly patient population. At our institution, every patient with degenerative mitral valve regurgitation is a candidate for mitral valve repair with robotic assistance apart from those exclusions previously mentioned like severe mitral annular calcification, severe peripheral arterial disease, etc. 

Compared to the traditional method of median sternotomy, there have been concerns about the cost associated with robotic cardiac surgery. Propensity-matched studies have demonstrated that total costs of robotic mitral surgeries are similar to those of sternotomy [14]. Although early direct costs, likely due to equipment, were higher in robotic mitral surgeries, there was a higher late indirect cost for surgeries performed via median sternotomy. This late increased indirect cost was due to increased length of stay, transfusions, and readmission rates. Additionally, robotic mitral valve surgery and mitral valve surgery via traditional sternotomy have been compared with meta-analysis, which demonstrated significantly shorter cross-clamp and bypass times with traditional sternotomy. There was shorter intensive care unit and hospital length of stay with robotic mitral valve surgery [15].

There are some notable differences that should be brought up between some other institutions who use the robotic platform as well as other minimally invasive approaches. Robotic technology is one of the platforms available for mitral valve repair, and through our experience, we have demonstrated that the platform is comparable to any other type of minimally invasive port-access mitral valve surgery. We are aware that, in Europe, the robotic platform has not taken off, and that published repairs with mini-thoracotomies have very similar outcomes [16,17]. We think that the robotic approach confers incredible visualization of the mitral valve, and it is reproducible and allows for every type of repair. It is also important to note that this procedure can be very painful for patients. This is likely due to nerve irritation due to the torque and motions of the robotic arms. For the recent several hundred cases, we have altered our analgesic approach with paravertebral catheters which stay in place while the chest tubes are in place and effectively control pain. We do not currently have experience with cryonerve ablation. 

Our surgical technique involves external aortic cross-clamping using a Chitwood clamp and insertion of an external aortic root catheter for cardioplegia and venting, which has previously been described and illustrated in detail [18]. Some other surgeons have used an endoaortic balloon catheter that delivers cardioplegia and also provides aortic root venting [19]. This has several advantages, including the ability to perform redo operations without the need to dissect the aorta. There are several notable scenarios where this can be more difficult. You cannot use the Endoballoon in larger diameter aortas, and there may be a higher risk of aortic dissection and thromboembolic events [20]. However, recent large studies comparing external aortic clamping and Endoballoon did not show significant differences in safety outcomes [21]. In some Endoballoon cases, surgeons must use the other femoral artery because one femoral artery may not be big enough to accept the perfusion cannula with the Endoballoon in a side arm of the cannula.

## 5. Conclusions

In our experience of almost 1500 cases, we have seen excellent durability of repair and excellent patient outcomes. The robotic platform also enabled us to train young surgeons due to the ability to maintain full control and visualization of the operative field at any moment. While we understand that there are other approaches using a right mini-thoracotomy for repair with similar results, we conclude that the robotic platform gives greater visualization, ability to perform any type of repair, patient acceptance and satisfaction, and ability to teach in a very safe way using the teaching console.

## Figures and Tables

**Table 1:** Patient characteristics.

	All *N* = 1412	Case ≤ 120 *N* = 120	Case > 120 *N* = 1292	*p*-Value
Age, years (IQR)	63 (54–70)	56 (50–64)	63 (55–70)	<0.01
Male sex (%)	922 (65.3)	77 (64.2)	845 (65.4)	0.78
Height, cm (IQR)	175 (166–180)	175 (168–180)	175 (165–180)	0.77
Weight, kg (IQR)	75.3 (63.7–86.7)	76.7 (64.9–87.3)	75.0 (63.5–86.6)	0.35
BMI, cm/m^2^ (IQR)	24.5 (22.1–27.5)	25.2 (22.3–28.0)	24.5 (22.1–27.5)	0.17
Race (%)	1409	120	1289	
White	1167 (82.8)	108 (90.0)	1059 (82.1)	0.03
Asian	110 (7.8)	6 (5.0)	104 (8.1)	0.29
Hispanic	45 (3.2)	1 (0.8)	44 (3.4)	0.17
Black	33 (2.3)	3 (2.5)	30 (2.3)	0.76
Other	55 (3.9)	2 (1.7)	53 (4.1)	0.32
Diabetes mellitus (%)	76 (5.4)	2 (1.7)	74 (5.7)	0.06
Hypertension (%)	703 (49.8)	50 (41.7)	653 (50.5)	0.06
Peripheral vascular disease (%)	17 (1.2)	2 (1.7)	15 (1.2)	0.65
Cerebrovascular disease (%)	53 (3.8)	4 (3.3)	49 (3.8)	1.00
Prior myocardial infarction (%)	39 (2.8)	3 (2.5)	36 (2.8)	1.00
Atrial fibrillation (%)	343 (24.3)	27 (22.5)	316 (24.5)	0.63
Dialysis dependence (%)	2 (0.1)	0 (0)	2 (0.2)	1.00
Mitral endocarditis (%)	35 (2.5)	0 (0)	35 (2.7)	0.07
Mitral annular calcification (%)	58 (4.1)	1 (0.8)	57 (4.4)	0.06
Tricuspid regurgitation ≥ moderate (%)	303 (21.5)	24 (20.0)	279 (21.6)	0.68
NYHA class III–IV (%)	183/1403 (13.0)	50/120 (41.7)	133/1283 (10.4)	<0.01
LVEF, % (IQR)	62 (60–66)	60 (60–65)	62 (60–66)	0.28
STS PROM, % (IQR)	0.46 (0.28–0.77)	0.33 (0.23–0.54)	0.47 (0.28–0.81)	<0.01
STS PROMM, % (IQR)	5.90 (4.26–8.47)	4.34 (3.43–6.21)	6.05 (4.41–8.75)	<0.01

**Table 2:** Operative characteristics.

	All *N* = 1412	Case ≤ 120 *N* = 120	Case > 120 *N* = 1292	*p*-Value
Non-elective procedure (%)	31 (2.2)	0 (0)	31 (2.4)	0.10
Cross-clamp time, minutes (IQR)	77 (66–93)	112 (103–130)	75 (65–88)	<0.01
CPB time, minutes (IQR)	118 (105–140)	153.5 (134.5–177.5)	116 (103–136)	<0.01
Concomitant procedures (%)				
Tricuspid surgery	81 (5.7)	0 (0)	81 (6.3)	<0.01
PFO closure	264 (18.7)	0 (0)	264 (20.5)	<0.01
LAA occlusion	864 (61.2)	0 (0)	864 (66.9)	<0.01
Atrial fibrillation ablation	292 (20.7)	24 (20.0)	268 (20.7)	0.57
Mitral leaflet prolapse (%)				0.05
Posterior	926 (65.6)	88 (73.3)	838 (64.9)	
Anterior or bileaflet	396 (28.0)	30 (25.0)	366 (28.3)	
Other etiology	90 (6.4)	2 (1.7)	88 (6.8)	
Mitral valve repair				
All patients (%)	1385 (98.1)	118 (98.3)	1267 (98.1)	1.00
Degenerative patients (%)	1232/1238 (99.5)	109/111 (98.2)	1123/1127 (99.7)	0.09
Residual mitral regurgitation > mild on intra-operative TEE (%)	11 (0.8)	0 (0)	11 (0.9)	0.61

CPB, cardiopulmonary bypass; PFO, patent foramen ovale; LAA, left atrial appendage.

**Table 3:** Operative outcomes.

	All *N* = 1412	Case ≤ 120 *N* = 120	Case > 120 *N* = 1292	*p*-Value
Operative mortality (%)	4 (0.4)	1 (0.8)	3 (0.2)	0.30
Reoperation for bleeding or cardiac reason (%)	36 (2.6)	9 (7.5)	27 (2.1)	<0.01
Renal failure (%)	9/1409 (0.4)	3/117 (2.6)	6/1292 (0.5)	0.03
Sepsis (%)	2 (0.1)	1 (0.8)	1 (0.01)	0.16
Prolonged ventilation or reintubation (%)	24 (1.7)	4 (3.3)	20 (1.6)	0.14
Cerebrovascular accident (%)	10 (0.7)	4 (3.4)	6 (0.5)	0.01
Permanent pacemaker implantation (%)	24 (1.7)	3 (2.5)	21 (1.6)	0.45
Length of stay, days (IQR)	5 (5–7)	6 (5–7)	5 (5–6)	0.02

## Data Availability

The data used in this study were from our institutional registry of patients, which is prospectively maintained.

## Supplementary Materials

The following supporting information can be downloaded at: https://drive.google.com/file/d/1VKrSM1ia8GZGGBDYP9P-Ok3uS0jXIHUa/view, Video S1: Mitral repair with triangular resection; Video S2: Mitral repair with chordal transposition; Video S3: Mitral repair with neochordae.
